# A Mouse Model for *Candida glabrata* Hematogenous Disseminated Infection Starting from the Gut: Evaluation of Strains with Different Adhesion Properties

**DOI:** 10.1371/journal.pone.0069664

**Published:** 2013-07-23

**Authors:** Ralitsa Atanasova, Adela Angoulvant, Maurel Tefit, Frédérick Gay, Juliette Guitard, Dominique Mazier, Cécile Fairhead, Christophe Hennequin

**Affiliations:** 1 Université Pierre et Marie Curie-Paris6, UMR S945, Paris,France; 2 AP-HP, Hôpital Bicêtre, Unité de Parasitologie-Mycologie, Le Kremlin-Bicêtre, France; 3 Université Paris-Sud 11, Institut de Génétique et Microbiologie, and CNRS UMR 8621, Orsay, France; 4 APHP, Groupe Hospitalier Pitié-Salpêtrière, Service de Parasitologie-Mycologie, Paris, France; 5 INSERM, U945, Paris, France; 6 AP-HP, Hôpital Saint Antoine, Service de Parasitologie-Mycologie, Paris, France; David Geffen School of Medicine at University of California Los Angeles, United States of America

## Abstract

Adhesion to digestive mucosa is considered a crucial first step in the pathogenicity of invasive *Candida* infections. *Candida glabrata* disseminated infections predominantly start from the gut. A mouse model of disseminated infection starting from the gut was set up. Hematogenous dissemination was obtained after a low-protein diet followed by a regimen of cyclophosphamide-methotrexate and an oral inoculation of the yeasts via the drinking water. The liver was the first organ infected (day 7 post-infection), and lethality was 100% at day 21 post-infection. This new mouse model was used to compare the mortality rate and fungal burden in deep organs induced by 5 strains exhibiting different levels of adhesion to enterocyte Caco-2 cells, as determined in a test on 36 *C. glabrata* strains. In this model, no statistical difference of lethality was demonstrated between the strains, and fungal burden varied in kidneys and lungs but without correlation with the level of adhesion to enterocytes. Further studies using the model developed here allow analysis of the crossing of the digestive mucosa by yeasts, and help relate this to yet-poorly understood adhesion phenotypes.

## Introduction

Invasive candidiases are life threatening opportunistic infections, the incidence of which has dramatically increased during the 1980’s [1]. While *Candida albicans* remains the leading cause of those infections, *Candida glabrata* has significantly emerged and now regularly ranks number two among the etiologic agents of the different clinical forms of candidiases [2], [3]. *C. glabrata* represents a serious therapeutic issue due to its natural resistance to azole derivatives and, probably due to delay in appropriate therapy initiation, some reports have described higher mortality rates for infections with *C. glabrata* than with *C. albicans* [4].

The origin of candidiasis is considered as mainly endogenous, since different *Candida* species can be commensals of human mucosae, notably the digestive mucosa [5]. *C. glabrata* may belong to the gut microbiota, and has been shown to be the most common species isolated from the rectum of patients admitted in Intensive Care Units [6]. Thus, maybe more so than for *C. albicans,* for which intravenous catheters represent another important portal of entry, the gut seems to be the predominant origin of *C. glabrata* bloodstream infections [7]. However, the mechanisms of translocation of yeasts from the gut to the bloodstream have only been investigated in a limited way. Indeed, different hypotheses have been proposed to describe the crossing of *C. albicans* through the intestinal mucosa, although the exact mechanism of this process is not yet clearly elucidated [8]. This ignorance may be due, in part, to the lack of an animal model mimicking the step of gut translocation. Indeed, most animal models involve intravenous injection of the inoculum, thus bypassing the crossing of the digestive mucosa. For many pathogens, *in vitro* models have shown that adhesion to host cells is a crucial primary step to cross cellular barriers [9]. Indeed, in *C. glabrata,* a large family of genes called *EPA* for epithelial adhesins, encoding surface glycoproteins, has been shown to be essential for the adhesion to different epithelial human cells originating from the larynx (HEp2) or ovary Lec2 cells [10]–[12], and is possibly involved in virulence, as tested in animal models [11]. Based on whole genome comparative analysis, we have, ourselves, recently observed that the number of copies of *EPA* genes is associated with pathogenicity within the *Nakaseomyces* clade, which encompasses *C. glabrata* (T. Gabaldon, T. Martin et al., submitted). However none of these conclusions have been tested in a model that necessitates crossing of the gut mucosa.

Thus, in this work, we first wanted to set up a mouse model of disseminated infection by *C. glabrata* starting from the gut, that may be used further to analyze the mechanisms that predispose to the crossing of the digestive mucosal barrier by *C. glabrata*. Once validated, this model was used to challenge mice with clinical strains belonging to the same clonal complex, and previously characterized to have either a low or a high adhesion level as tested on the Caco-2 enterocyte cell line.

## Materials and Methods

### Strains

A panel of 36 *C. glabrata* clinical strains, plus the reference strain ATCC2001 (CBS138), was used in this study. Their genotype and clonal group classification had been previously determined using a molecular method based on the analysis of length polymorphism of microsatellite containing regions [13], [14]. The mating type of each of these strains had also been previously determined using a combination of specific primers. For this study, strains were selected so as to represent at the best the genotypic diversity of the species (i.e. belonging to the different clonal complexes from above mentioned studies). A monoclonal subculture of each strain was stored at −80°C. For experiments, stock cultures were plated on 1% yeast extract 1% peptone-2% glucose (YPD) agar and then incubated at 37°C for 24 h.

### Murine Model of Hematogenous Disseminated Candidiasis

To correlate *in vitro* adhesion with *in vivo* virulence, we set up a murine model of *C. glabrata* hematogenous dissemination starting from the gut. Animals were first subjected to a low-protein diet of varying length. Infection was then performed via the oral route; and an immunosuppressive regimen combining methotrexate (MTX) plus cyclophosphamide (CPA) was started. During preliminary experiments, we tested several factors that may potentially affect the course of the infection. Notably we compared the age of animals (3 versus 5 weeks), the duration of the low-protein diet (2 weeks versus 3 weeks) and the immunosuppressive drugs’ regimens (3 vs 5 consecutive days of CPA administration). Female DBA/2 J mice (Centre d’Elevage Charles River, L’Abresle, France) were used for all experiments. Animals were housed in groups of six (preliminary experiments) or eight (adhesion experiments) in filter-top cages with free access to food and water. After one week of acclimation, mice were subjected to a low-protein diet containing 5% casein (SAFE, Augy, France). Animals were then starved for 24 hours before infection, which consisted in administration of contaminated drinking water ad libitum for five days. Water was inoculated with yeasts harvested from 48 hour-cultures on YPD at 37°C, washed twice in sterile PBS and suspended to a final concentration of 5×10^7^ CFU/ml. Digestive colonization was controlled at day -1 and at day 2 before and after infection, by plating stool samples from each animal. At day 4 post-infection, contaminated water was switched for sterile water complemented with enrofloxacin (80 mg/ml) (Bayer, France), a fluoroquinolone antibiotic. Meanwhile, immunosuppressive therapy with MTX (0.15 mg/g of body weight) and CPA (0.15 mg/g of body weight) (Sigma-Aldrich, France) was initiated. Mice were monitored daily for morbidity for 21 days after infection. Moribund mice were euthanized, as well as surviving animals at the end of the experimental protocol. Their livers, kidneys and lungs were aseptically removed, weighted and extensively homogenized in 0.5 ml sterile PBS. Dilutions were plated on CHROMagar medium (Becton-Dickinson, Le Pont de Claix, France) and incubated at 37°C for 48–72 h before CFU counting. Blood was also collected by cardiac puncture in mice under terminal anesthesia. Two hundred and fifty µl of blood sample were inoculated in 10 ml Sabouraud medium and incubated 48 h at 37°C prior to subculture on CHROMagar plates. To compare the dynamics of infection, three groups of six animals were followed with euthanasia of one animal from each group every day, starting from day 2 after the initiation of chemotherapy regimen and until day 12 after infection.

The study was carried out in accordance to European legislation covering the use of animals for scientific purposes. The protocol was approved by the local Institutional Animal Care and Use committee of the Faculté de Médecine Pitié-Salpêtrière.

### Enterocyte Adhesion

Adhesion assays were performed using the Caco-2 cell line, derived from a colorectal carcinoma [15]. Cells were grown onto 11 mm diameter glass coverslips placed in 24-well plastic dishes. Cells were allowed to differentiate in monolayers for 16–21 days at 37°C with 5% CO2. Cells were washed and infected with 10^7^ stationary growth phase *C. glabrata* cells suspended in 500 µl of pre-heated phosphate buffered saline (PBS). After a 30 min-incubation period at 37°C, the cells were gently washed three times to remove non-adherent yeasts. Caco-2 cells with adherent yeasts were then scraped and collected in 500 µl of PBS. Serial dilutions were plated onto YPD medium and incubated for 48 hours at 37°C. Colony forming units (CFUs) were counted and used to calculate the adhesion rate as the ratio of CFUs divided by the number of inoculated cells (10^7^). In order to limit the impact of inter-assay variability, adhesion rate of a given strain was divided by the adhesion rate of ATCC2001 reference strain tested in the same experiment. Each isolate was tested in duplicate, and two separate experiments were performed (four datasets collected).

### Statistical Analysis

Statistical analysis was performed using GraphPad Prism vs 5.0 (GraphPad software). Medians of repeated measures of adhesion to Caco-2 cells were compared between strains using the Kruskal-Wallis test followed by Dunn’s post-test for each pair of strains. Similarly, comparisons between clonal complexes and between mating type were performed using the Kruskal-Wallis rank sum test (quantitative scale). Comparison of survival curves was done using the Mantel-Cox log rank test. Fungal burden in organs as determined by CFU counts per g was analyzed with Mann-Whitney’s non-parametric test when two groups were compared, and with Kruskal-Wallis’ non-parametric test for more than two groups. Pairs of groups were further compared with Dunn’s test. A *P* value of less than 0.05 was considered significant.

## Results

### Development of a Mouse Model of Hematogenous Candidiasis with the Gut as Starting Point

In order to mimic at best the hematogenous dissemination of *C. glabrata* starting from the gut, we developed a mouse model with oral inoculation of yeasts, using the ATCC2001 type strain. Before infection, mice were fed with a low-protein diet, a predisposing factor previously shown to thin down the mucosa of the small intestine and colon, favoring *Candida* colonization [16]. Neutropenia was further induced by a cytotoxic chemotherapy regimen.

**Figure 1:**
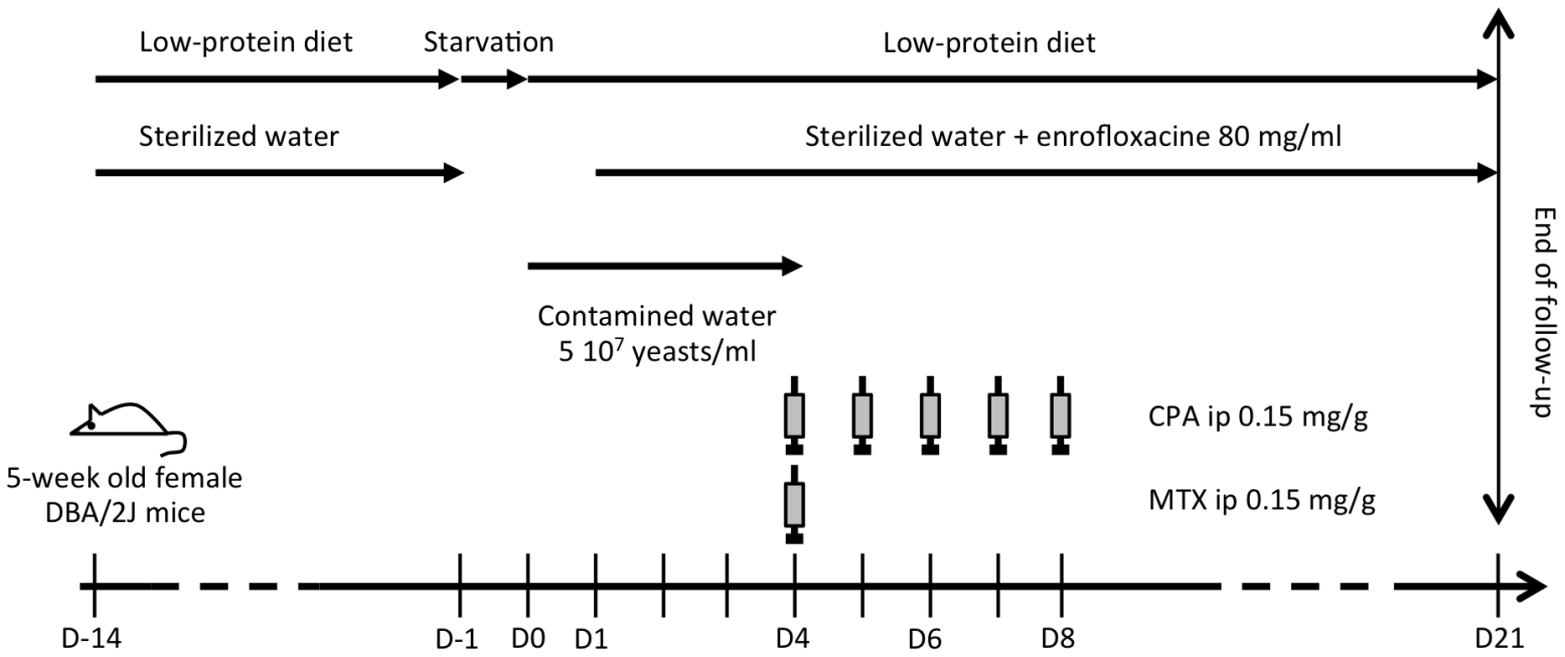
Experimental protocol of *C. glabrata* colonization and dissemination in animal model. Five weeks old female DBA2/J mice are infected with 5×10^7^ CFU/ml in drinking water for five days after 14 days of low-protein diet and 24 h starvation. A chemotherapy protocol including a single MTX ip injection and an injection of CPA/day for five consecutive days follows the infection. Mice are provided with sterile water supplied with enrofloxacine from the beginning of chemotherapy until the end of experiment. MTX: methotrexate, CPA: cyclophosphamide.

**Figure 2:**
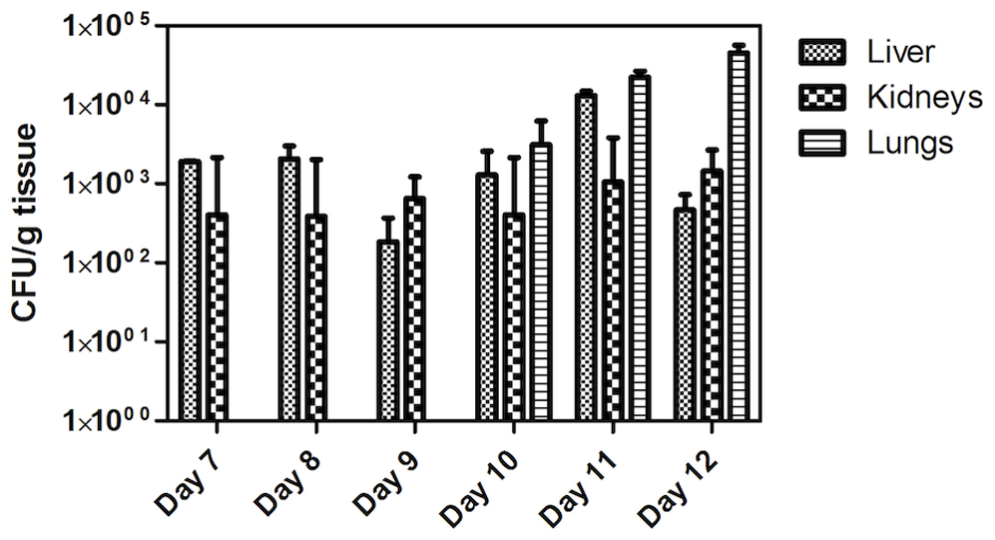
Kinetics of *C. glabrata* dissemination in organs of infected mice. Mice were housed in three groups of 6 animals. After sacrifice of an animal from each group at different time points after infection, organs were aseptically removed and homogenized for CFU count (n = 3/day).

A number of factors affecting the mortality rate and the fungal burden in the targeted organs were first analyzed. We found that non-specific mortality (death with the lack of fungal involvement of organs as examined by culture of ground organs) was lower in 5-weeks than in 3-weeks old mice: 20–30% versus 90% (data not shown). We also demonstrated that a 14-days diet was sufficient to implement a gut *Candida* colonization with subsequent dissemination, without significant difference for survival rate or organs’ infection as compared to a 21-days diet (Supplementary data Figure S1A–B). In contrast, 3 consecutive injections of CPA, while leading to a similar mortality rate, resulted in a lesser fungal burden in the organs as compared to a 5 injections protocol (Supplementary data Figure S1A–C). Based on these preliminary data, we designed an optimized experimental protocol that consists of a 2 week-long low-protein diet prior to infection with 5×10^7^ CFU/ml yeasts suspended in drinking water, followed by an individual intraperitoneal injection of MTX on day 5 post-infection and 1 CPA injection/day for five consecutive days (Figure 1). Using this protocol, a mortality rate of 60±20% was observed between days 14 and 16 post-infection and 100% at days 18–21 post-infection. A remaining 5 to 20% non-specific mortality rate was observed in non-infected animals, mostly due to bacteremia (data not shown). Analysis of infection dynamics showed that *C. glabrata* was first recovered from liver and kidneys as early as 7 days post-infection, then from the lungs three days later (Figure 2). Mean fungal burden in liver decreased from 2×10^3^ CFU/g at day 7 to 0.05×10^3^ CFU/g at day 12. In contrast, fungal burden in kidneys increased from 0.04×10^3^ CFU/g at day 7 to 1.4×10^3^ CFU/g at day 12. Absence of yeasts in the lungs was noticed until day 10 after infection, with a mean value increasing from 0.3×10^4^ CFU/g to 4.5×10^4^ CFU/g at the end of the experiment. Blood cultures remained negative in all cases, except for three samples drawn at day 10 (1 case) and day 12 (2 cases) post-infection.

### Adhesion to Digestive Epithelial Cells Differs between Strains but is not Clade-dependent

**Figure 3:**
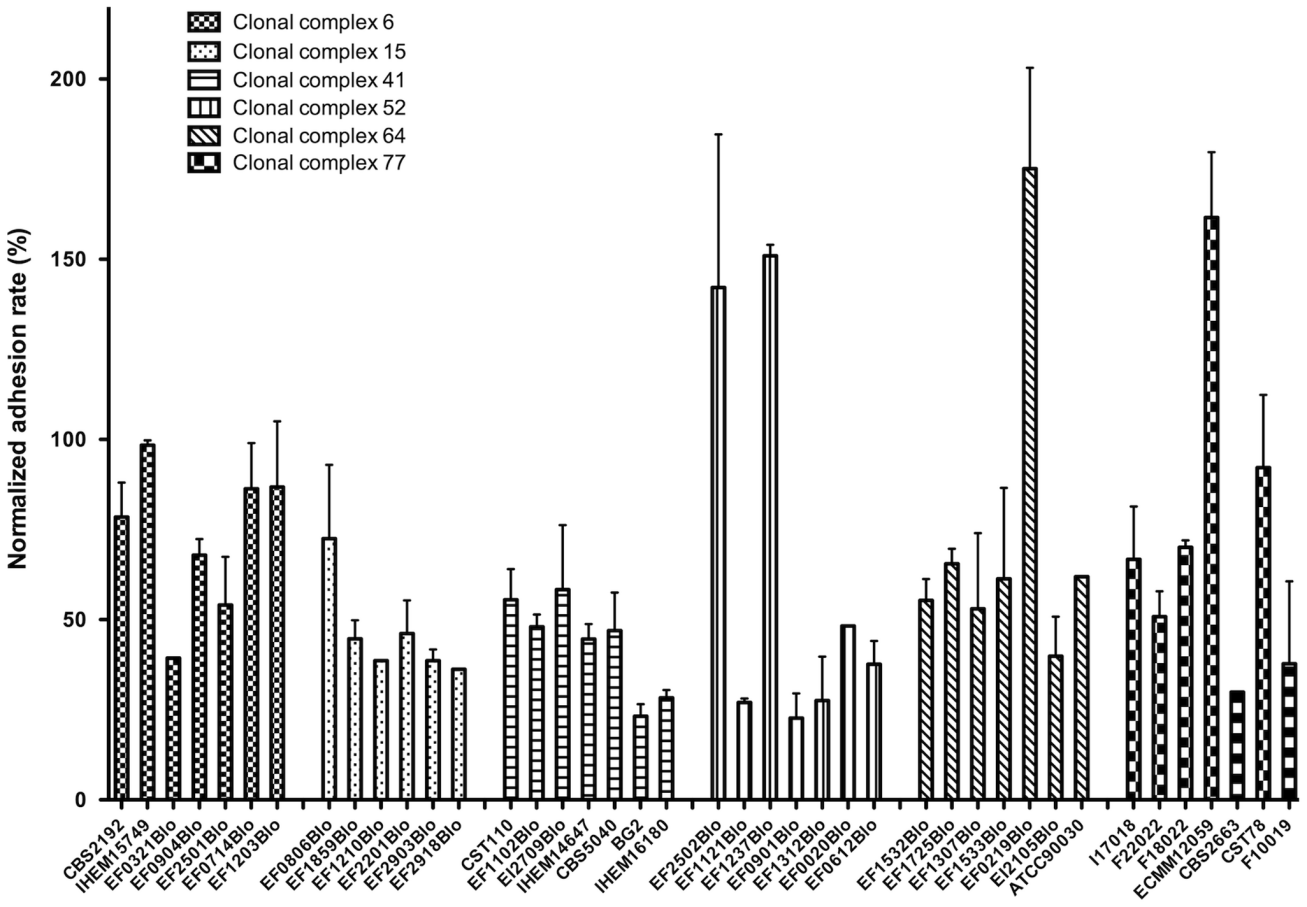
Adhesion rate of *C. glabrata* strains to Caco-2 cells. Adhesion level rate is expressed in percentage after normalization to ATCC2001 reference strain adherence level used in each experiment as a control. Results are presented based upon the clonal complex belonging as previously determined [13], [14]. Strains EF0806Blo, EF1859Blo, EF2918Blo, EF1210Blo, EF0904Blo, EF2501Blo are of alpha mating type. The mating type of strains EF2502Blo, EF0612Blo, EF2903Blo, IHEM15749 have not been determined. Other strains are of A mating type.

Adhesion assays on Caco-2 cells were performed for 36 selected clinical *C. glabrata* strains whose genotype and mating type had been previously determined. Important differences in adhesion level were observed between strains with values ranging from 23% (strain EF0901Blo) to 175% (strain EF0219Blo) as compared to the reference ATCC 2001 strain (100%) (Figure 3). There was a statistical difference between the median values of adhesion of strains (Kruskall Wallis statistic 127.4; p<0.0001). Significant differences between pairs of strains found using Dunn’s post-test are summarized in Table 1. However, it was not possible to demonstrate any significant difference between clades (Kruskal-Wallis statistic 4.297; p-value: 0.51) with non-significant Dunn’s multiple comparison tests (p>0.05). Similarly, no difference was noted between *MATa* (n = 26) and *MATalpha* (n = 6) strains (Mann-Witney test 80.00; p>0.6438).

**Table 1:** Pairs of *C. glabrata* strains with significant difference in adhesion to Caco-2 cells as tested with the Dunn’s post-test.

Pairs of strains	Difference in rank sum	P value
IHEM15749 (CC77) vs BG2	117	<0.05
BG2 vs EF2502Blo	−127	<0.05
BG2 vs EF1237Blo	−127	<0.05
BG2 vs EF0219Blo	−133	<0.01
BG2 vs ECMM12059	−129	<0.01
IHEM16180 vs EF2502Blo	−118	<0.05
IHEM16180 vs EF1237Blo	−118	<0.05
IHEM16180 vs EF0219Blo	−124	<0.05
IHEM16180 vs ECMM12059	−120	<0.05
EF2502Blo vs EF1121Blo	120	<0.05
EF2502Blo vs EF0901Blo	126	<0.05
EF2502Blo vs EF1312Blo	118	<0.05
EF1121Blo vs EF1237Blo	−120	<0.05
EF1121Blo vs EF0219Blo	−126	<0.05
EF1121Blo vs ECMM12059	−122	<0.05
EF1237Blo vs EF0901Blo	126	<0.05
EF1237Blo vs EF1312Blo	118	<0.05
EF0901Blo vs EF0219Blo	−132	<0.01
EF0901Blo vs ECMM12059	−128	<0.01
EF1312Blo vs EF0219Blo	−124	<0.05
EF1312Blo vs ECMM12059	−120	<0.05
EF0219Blo vs CBS2663	118	<0.05

### Morbidity and Mortality are not Correlated with *in vitro* Adhesion

In order to investigate the possibility of a correlation between adhesion to epithelial cells and *in vivo* pathogenicity, five strains were selected for *in vivo* testing using the animal model previously developed. To limit the potential effect of clonal complexes, four strains exhibiting different levels of adhesion were selected within the same clonal complex (Figure 3). Since none of these had adhesion levels similar to strain ATCC2001, we included a fifth strain (IHEM15749) with this property.

**Figure 4:**
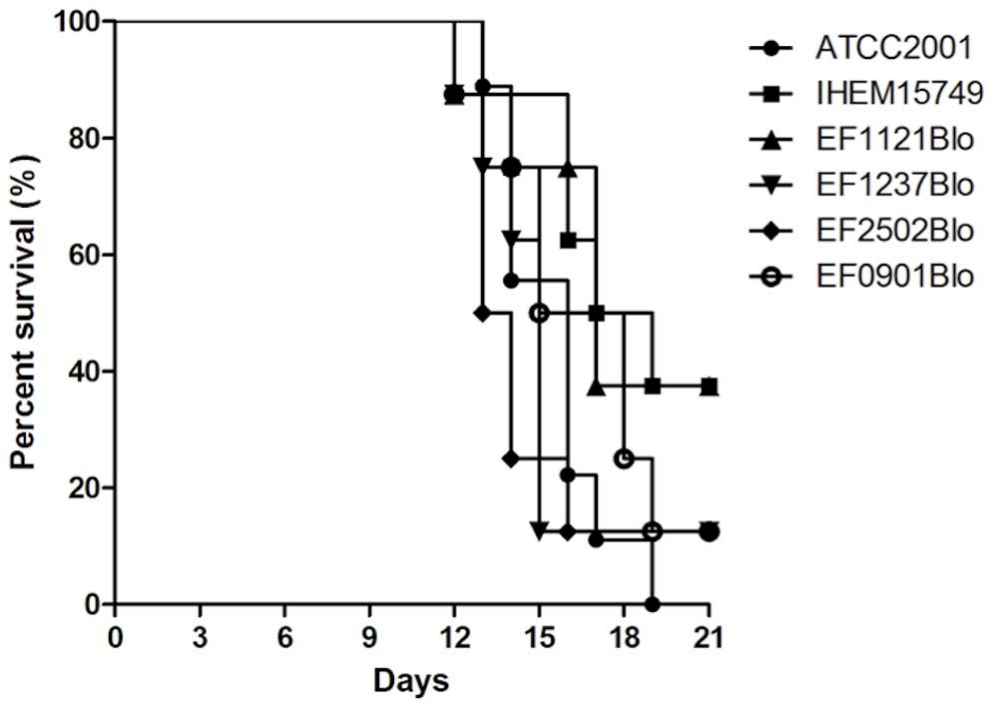
Survival curves for mice infected with six different *C. glabrata* strains. Adhesion levels of the strains used for the infection are as follows: 100% for ATCC2001, 98.43% for IHEM15749, 27% for EF1121Blo, 151% for EF1237Blo, 142% for EF2502Blo and 23% for EF0901Blo. No statistically significant differences in survival were determined using the Mantel-Cox log rank test (Prism 5.0).

The mortality rate at day 21 post-infection varied between 32.5% (IHEM15749 and EF1121Blo) and 100% (ATCC2001) (Figure 4). Yeasts were isolated from at least one organ for each animal. Analysis of the Kaplan-Meier curves did not show significant difference between strains (Mantel-Cox log rank test, Prism 5.0).

**Figure 5:**
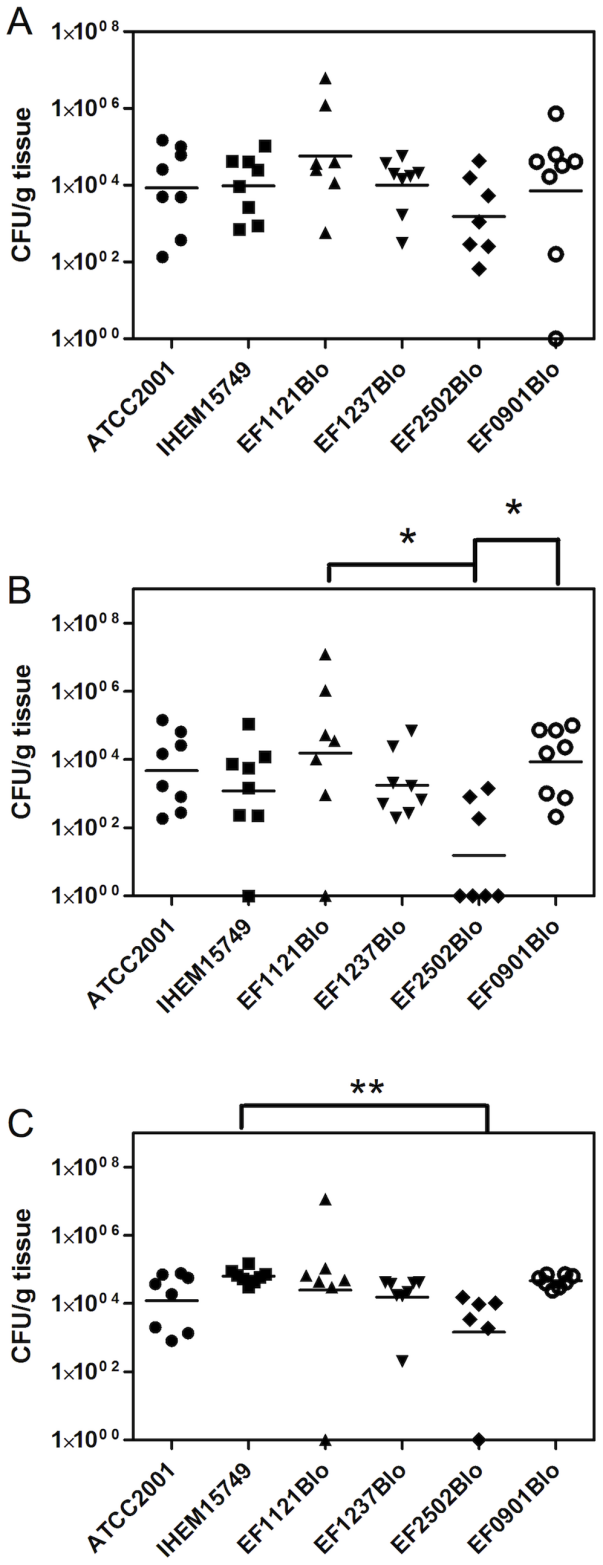
Comparison of fungal tissue burden following infection of mice with six different *C. glabrata* strains. Dissemination in liver (a), kidneys (b) and lungs (c) was determined after sacrifice and aseptically removal of organs from moribund mice. Dissemination rate expressed as CFU/g tissue was compared between groups by Kruskal-Wallis non-parametric test followed by Dunn’s post-test (Prism 5.0). Statistically significant differences were observed in kidneys’ fungal burden between EF2502Blo strain compared to EF1121Blo and EF0901Blo strains (*, *P*<0.05) and in lungs between EF2502Blo and IHEM15749 (**, *P*<0.005).

There was no statistical difference between the strains concerning the fungal burden in the liver (Kruskal-Wallis non-parametric test, followed by Dunn’s post-test). However, the strain EF2502Blo tended to induce a lower fungal burden in the other organs (Figure 5). This difference reached statistical signification for kidneys when compared to the fungal burden induced by strains EF0901Blo and EF1121Blo, and lung burden when compared to IHEM15749 (Dunn’s test; P<0.005).

## Discussion

*C. glabrata* represents a major threat in many medical institutions due to its increasing frequency and its natural antifungal resistance to azoles, as well as its acquired resistance to echinocandins, as recently reported [2], [17], [18]. Molecular typing studies support the idea that the gut is the main portal of entry of *C. glabrata* bloodstream infections [19]. However, the *in vivo* study of *C. glabrata* pathogeny remains a challenge since almost all animal models of disseminated *C. glabrata* infection use intravenous injection of yeasts [20]–[22], bypassing the crucial step of gut translocation. Recent studies have demonstrated that dextran sodium sulfate (DSS)-induced colitis may favor the gastro-intestinal colonization with either *C. albicans* [23] or C. *glabrata* [24]. Neutropenia, induced by cytotoxic chemotherapy or selective neutrophil depletion using specific monoclonal antibodies, may then ensure the dissemination of *C. albicans* [23]. However, few patients with disseminated infection exhibit such highly inflammatory lesions. In contrast, malnutrition is common in cancer and elderly patients who are frequently colonized and infected with *C. glabrata* [3], [6]. Our results show that, as previously reported for *C. albicans* [25], a low protein diet together with an antibiotic treatment, mimic, in mice, the factors that favor the implantation of *C. glabrata* in the gut. Subsequent immunosuppressive therapies then enables dissemination, which first targets the liver, in accordance with the draining vessels of the starting point of dissemination, while kidneys and lungs are involved later in the evolution of infection. One can note that the use of MTX in the cytotoxic regimen may participate to the digestive mucosal damage and act as an additional factor favoring gut translocation.

In order to test our model with strains isolated from clinical patients, after its validation with the type strain, we selected 5 strains characterized by various level of adhesion to the Caco-2 enterocyte cell line. These clinical strains were chosen within the same clonal complex in order to limit genotypic and phenotypic variability. Another strategy could have been to study *in vitro* generated mutants such as those deleted for cluster(s) of *EPA* genes [26], keeping in mind that such strains are never isolated from clinical sources. Indeed we have genotyped by specific PCRs one hundred *C. glabrata* strains for the genomic contents of some of their EPA genes (*EPA1*, *EPA2*, *EPA4* and *EPA6*) and never observed major loss of these genes in any strain. Although variation in the presence/absence of individual genes exists, polymorphism mostly concerns the length of internal repeats within the genes, giving rise to large paralogous protein families with numerous repeat variation (data not shown). When the 5 selected strains were used to challenge our mouse model, we did not notice any significant difference of mortality. In contrast, we observed a significantly lower fungal burden, particularly in kidneys and lungs, for strain EF2501Blo, which exhibited the highest adhesion rate. The fact that conversely the strain EF1237Blo, which was characterized by a similar level of adhesion to Caco-2 cells, was associated with a high fungal burden in the organs may suggest that adhesion to Caco-2 cells does not reflect *in vivo* adhesion to murine enterocytes. Indeed, this is a limit of our study in which we have tested *in vitro* adhesion onto human enterocytes whereas virulence was evaluated in a mouse model. It is known that cell-surface receptors may differ from human to mouse and thus may impact the results. Moreover, an inherent bias of the Caco-2 cell line is that these cells do not produce mucus, thus facilitating the contact between yeasts and the host cells. It would be interesting to compare adhesion on different types of human and murine tissues, since it has already been demonstrated that adhesion and invasion are not only pathogen-dependent but also depend on the cell type and its level of differentiation [27].

Alternatively, one could hypothesize that other factors, either from the host or the pathogen, are involved in bloodstream dissemination. Studies looking at a potential correlation between *Candida in vitro* adhesion and *in vivo* virulence are relatively scarce. The adhesion of *C. glabrata* to various epithelial and endothelial cells has been described [28], [29], but studies focusing on adhesion of *C. glabrata* to intestinal cells are relatively scarce [30], to our knowledge.

Moreover, while the *EPA1* lectin was first shown to be required for efficient *in vitro* adhesion to human laryngeal cells, its deletion did not correlate with decreased virulence in a model of mucosal infection [12]. Using an intravenous systemic infection animal model, De Las Penãs et al further demonstrated that deleting 4 genes (*HYR1*, *EPA1*, *EPA2*, and *EPA3*) in the *EPA1* cluster led to a limited (3- to 5- fold), though significant, reduction of kidney (but not spleen nor liver) infection, supporting the idea that these genes may play a role in virulence, albeit a modest one [26]. It is difficult to conclude whether this lesser virulence resulted from a deficiency in epithelial adhesion *in vivo,* since this step was by-passed by the direct intravenous injection of the yeasts. Since the role of *EPAs* in the adhesion to endothelial cells and in phagocytosis by macrophages has been demonstrated [29], [31], an alternative hypothesis is that these mechanisms predominate over the role of *EPAs* in the adhesion to epithelial cells.

Here, we demonstrate that *in vitro* adhesion to intestinal cells varies according to fungal strains, but we were unable to correlate adhesion level with either mating type, as it has been well documented for the pathogenic basidiomycetous yeast *Cryptococcus neoformans* [32], or with microsatellite-based genotype (belonging to a given clonal complex). Similarly, recent data in *C. albicans,* shows the lack of correlation between genotype and the commensal or clinical origin of the isolates [33]. Genotyping genes potentially involved in epithelial adhesion, such as *EPA* genes or β-mannosyl-transferases, may be more relevant. It has recently been shown that *C. glabrata* strains put in contact with host cells (Hep-2 epithelial cells) exhibit a great variation in *EPA1* expression [34], so looking for a correlation between transcriptomic profile and *in vivo* virulence should also be considered as a tool for a better understanding of the role of these genes in virulence.

Keeping in mind these limits, it can also be speculated that intestinal adhesion is not by itself essential for the occurrence of bloodstream dissemination. Recognition of the yeasts by the immune cells of the gut, such as dendritic cells or possibly enterocytes, is obviously important to develop an immune response that allows the yeasts to persist in the digestive tract without generating an inappropriate high inflammatory response [35]. In contrast, invasive infection with *C. glabrata* may result rather from a “passive” gut translocation favored by mucosal damage and the possibility of crossing between epithelial cells that may be loosened or absent. The lack of, or alteration in, yeast recognition by epithelial cells may also limit the activation of the immune response, which, together with the chemotherapy-induced neutropenia, may favor local infection and subsequent hematogenous dissemination. Similarly to microbial translocation, the alteration of the normal gastrointestinal microbiological equilibrium, increased permeability of mucosal barrier and immune system deficiencies seem to play a crucial role [36], [37].

In conclusion, we have set up an original mouse model of *C. glabrata* disseminated infection starting from the gut that mimics at best human infection. This model should be a powerful tool for further analyses of fungal virulence traits.

## Supporting Information

Figure S1 **Impact of low protein diet duration and immunosuppressive chemotherapy protocol on the outcome in an animal model of** ***C. glabrata*** **hematogenous dissemination.** Analysis of immunosuppressive protocols was done with animals fed with low-protein diet for 14 days. Comparative analysis of the 2 low-protein diets was performed with a 5-days CPA protocol. A: Survival rates of mice infected with *C. glabrata* ATCC2001 from two separate experiments. No statistical difference between groups were determined by Mantel-Cox log rank test (Prism 5.0). B: Tissue burden in organs of mice after 14 or 21 days of PCM prior to infection. No statistical difference between groups was observed (Mann-Whitney non-parametric test, Prism 5.0). C: Tissue burden in organs of infected mice rendered neutropenic after either 3 or 5 CPA ip injection (Mann-Whitney test, * *P*<0.05). CPA: cyclophosphamide
(TIF)
