# Supplementary figures and images for: A Mouse Model for Candida glabrata Hematogenous Disseminated Infection Starting from the Gut: Evaluation of Strains with Different Adhesion Properties
Source: PLoS One. 2013 Jul 23;8(7):e69664. doi: 10.1371/journal.pone.0069664 (PMC3720583; doi:10.1371/journal.pone.0069664)

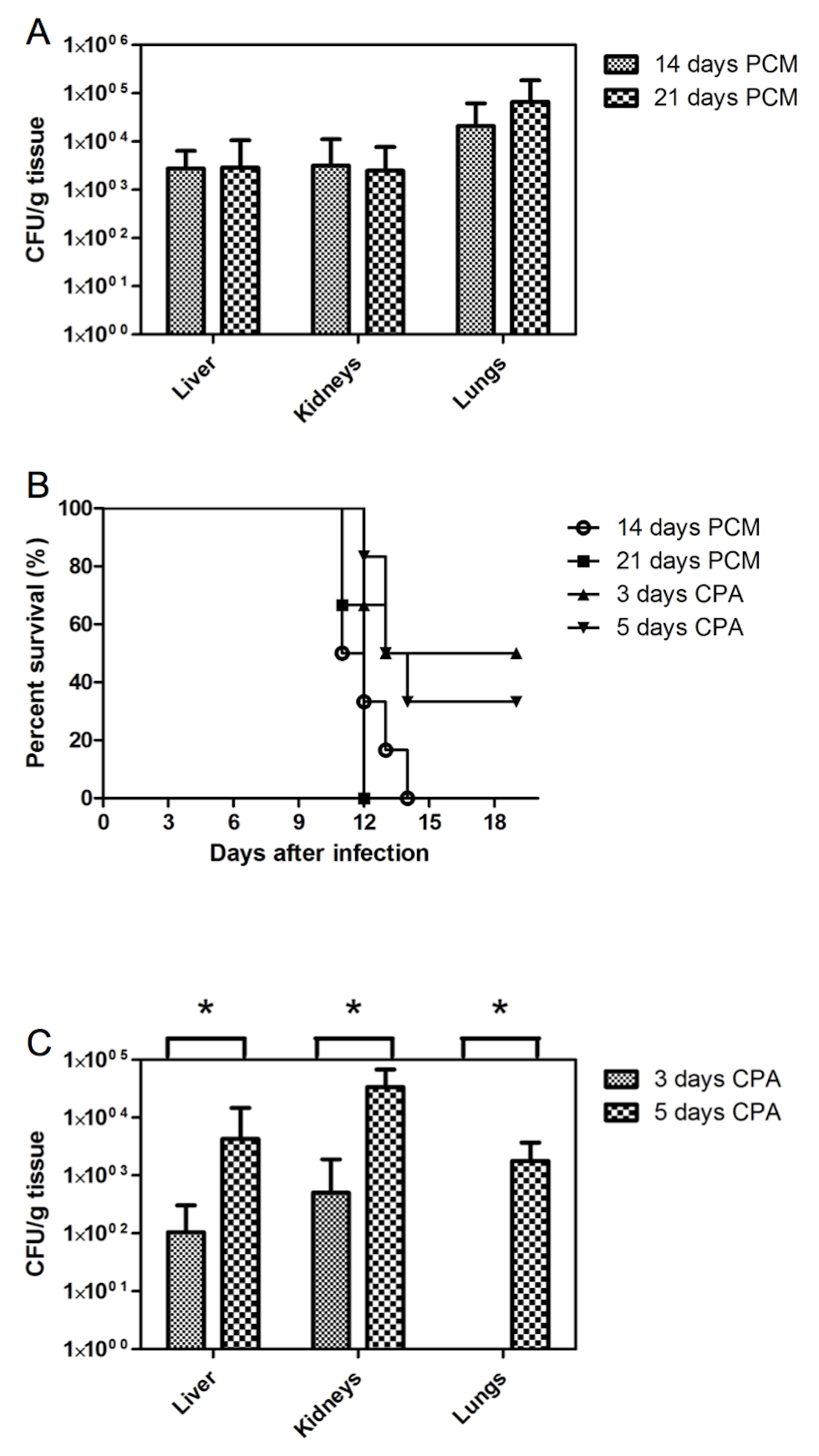

Supplement: Figure S1 — Impact of low protein diet duration and immunosuppressive chemotherapy protocol on the outcome in an animal model of C. glabrata hematogenous dissemination. Analysis of immunosuppressive protocols was done with animals fed with low-protein diet for 14 days. Comparative analysis of the 2 low-protein diets was performed with a 5-days CPA protocol. A: Survival rates of mice infected with C. glabrata ATCC2001 from two separate experiments. No statistical difference between groups were determined by Mantel-Cox log rank test (Prism 5.0). B: Tissue burden in organs of mice after 14 or 21 days of PCM prior to infection. No statistical difference between groups was observed (Mann-Whitney non-parametric test, Prism 5.0). C: Tissue burden in organs of infected mice rendered neutropenic after either 3 or 5 CPA ip injection (Mann-Whitney test, * P<0.05). CPA: cyclophosphamide (TIF) [file pone.0069664.s001.tif]
